# Detailed monitoring of a small but recovering population reveals sublethal effects of disease and unexpected interactions with supplemental feeding

**DOI:** 10.1111/1365-2656.12348

**Published:** 2015-03-09

**Authors:** Simon Tollington, Andrew Greenwood, Carl G. Jones, Paquita Hoeck, Aurélie Chowrimootoo, Donal Smith, Heather Richards, Vikash Tatayah, Jim J. Groombridge

**Affiliations:** ^1^ Durrell Institute of Conservation and EcologyUniversity of KentCanterburyCT2 7NZUK; ^2^ Mauritian Wildlife FoundationGrannum RoadVacoasMauritius; ^3^ International Zoo Veterinary GroupStation House, Parkwood StreetKeighley, West YorkshireBD21 4NQUK; ^4^ Durrell Wildlife Conservation TrustLes Augres ManorTrinityJerseyJE3 5BPUK; ^5^ Institute for Conservation ResearchSan Diego Zoo Global15600 San Pasqual Valley RoadEscondidoCAUSA

**Keywords:** disease ecology, ecological immunology, generalized linear mixed models, psittacine beak and feather disease, reproductive success, supplementary feeding

## Abstract

1. Infectious diseases are widely recognized to have substantial impact on wildlife populations. These impacts are sometimes exacerbated in small endangered populations, and therefore, the success of conservation reintroductions to aid the recovery of such species can be seriously threatened by outbreaks of infectious disease. Intensive management strategies associated with conservation reintroductions can further compound these negative effects in such populations.
2. Exploring the sublethal effects of disease outbreaks among natural populations is challenging and requires longitudinal, individual life‐history data on patterns of reproductive success and other indicators of individual fitness.
3. Long‐term monitoring data concerning detailed reproductive information of the reintroduced Mauritius parakeet (*Psittacula echo*) population collected before, during and after a disease outbreak was investigated.
4. Deleterious effects of an outbreak of beak and feather disease virus (BFDV) were revealed on hatch success, but these effects were remarkably short‐lived and disproportionately associated with breeding pairs which took supplemental food. Individual BFDV infection status was not predicted by any genetic, environmental or conservation management factors and was not associated with any of our measures of immune function, perhaps suggesting immunological impairment. Experimental immunostimulation using the PHA (phytohaemagglutinin assay) challenge technique did, however, provoke a significant cellular immune response.
5. We illustrate the resilience of this bottlenecked and once critically endangered, island‐endemic species to an epidemic outbreak of BFDV and highlight the value of systematic monitoring in revealing inconspicuous but nonetheless substantial ecological interactions. Our study demonstrates that the emergence of such an infectious disease in a population ordinarily associated with increased susceptibility does not necessarily lead to deleterious impacts on population growth and that negative effects on reproductive fitness can be short‐lived.

## Introduction

The impact of infectious diseases on the demographic parameters of host populations has become an established component of small population biology (McCallum & Dobson 1995; Hudson *et al*. 2002; De Castro & Bolker 2005; Lloyd‐Smith *et al*. 2005). Congruent with these regulatory effects predicted by ecological theory, the success of many high‐profile species reintroduction programmes has been challenged by the threat of novel or endemic infectious diseases. Indeed, many of the most iconic reintroduction projects including that of the black‐footed ferret (*Mustela nigripes*) (Thorne & Williams 1988), the pink pigeon (*Nesoenas mayeri*) (Swinnerton *et al*. 2005), the grey wolf (*Canis lupus*) (Almberg *et al*. 2012) and the Canadian lynx *(Lynx canadensis*) (Wild, Shenk & Spraker 2006) have been interrupted by the effects of infectious diseases. Consequently, identifying the potential impacts of infectious diseases in reintroduced populations is becoming increasingly important (Ballou 1993; Cunningham 1996; Ewen *et al*. 2012; Sainsbury & Vaughan‐Higgins 2012). However, quantifying and predicting their effects can be a challenge; negative impacts of disease are often not apparent until many years after a population is initially exposed (Ewen *et al*. 2007; Raisin *et al*. 2012). Nevertheless, since such populations are often the focus of detailed monitoring, they can prove highly informative for refining our understanding of infectious disease.

Importantly, small population effects underpin many aspects of disease dynamics in reintroduction biology. Small populations (a trait of most reintroduced populations) are characterized by reduced genetic diversity and increased levels of inbreeding (Lande 1988; Frankham 1997; Keller *et al*. 2001; Taylor & Jamieson 2008). In turn, these effects can lead to increased disease susceptibility (Acevedo‐Whitehouse *et al*. 2003; Spielman *et al*. 2004). Reduced population size and fragmentation among endangered species can disrupt host–parasite relationships, reduce herd immunity and result in the local elimination of endemic diseases as population fragments decline in size beyond a threshold required to maintain viral transmission (Lyles & Dobson 1993; Cunningham 1996). Disrupting density‐dependent host–parasite relationships in this way can leave local populations immunologically maladapted to novel strains of endemic diseases (Lyles & Dobson 1993), leaving populations even more prone to the adverse effects of subsequent epidemics (Cunningham 1996). This additional fragility may not be apparent until an endangered host population is recovered to a threshold size or density, through conservation intervention for example, which can subsequently increase the risk of novel epidemics (Cunningham 1996).

Establishing persistent wildlife populations via reintroductions is therefore not straightforward, particularly given the need to ensure appropriate measures to prevent disease outbreaks affecting the wider ecological community. An integral facet of any species' conservation strategy is the mitigation of disease risks, but it is often the case that a greater priority is immediate intensive management to increase population size. This hands‐on approach to species' recovery, which may involve translocations and maintaining individuals at high densities in captive breeding programmes, can potentially exacerbate the negative effects of disease. A trade‐off therefore exists between conservation intervention intended to maintain or increase species' numbers and mitigating the disease risk of that management. Balancing this trade‐off is a challenge central to the success of many conservation projects highlighted by the urgency of interventive action required to prevent the spread of facial cancer in Tasmanian devils (*Sarcophilus harrisi*) (Jones *et al*. 2007), the strategy to mitigate disease risk whilst maintaining population genetic viability by translocating hihi (*Notiomystis cincta*) in New Zealand (Parker, Brunton & Jakob‐Hoff 2006) and the threat of chytridiomycosis to the success of amphibian recovery efforts (Walker *et al*. 2008). Nevertheless, the popularity of reintroduction as a tool for species conservation continues and many of these recovery initiatives can expect to be interrupted by the negative effects of disease.

The case of the Mauritius (or echo) parakeet (*Psittacula echo*) represents an example of a reintroduction programme that was interrupted by the outbreak of disease and has been the focus of a long‐term intensive monitoring protocol resulting in detailed life‐history data. Once found throughout Mauritius, forest destruction in the 19th and 20th centuries reduced the population to fewer than 20 individuals which existed in several small, isolated pockets of natural forest in the 1980s (Jones & Duffy 1993). Population numbers were initially bolstered in the 1990s by the development of an intensive conservation strategy consisting of captive breeding, hand‐rearing and the manipulations of wild broods which included rescuing underdeveloped chicks from failing nests. The species was reintroduced to parts of its former native range in a series of releases between 1997 and 2005. In total, 139 hand‐reared juveniles (either captive‐bred or rescued from the wild) were released to augment the wild population. The simultaneous introduction of both artificial nest boxes and supplemental feeding through provision of food supplements at designated feeding stations further promoted population growth and continues to do so (Tollington *et al*. 2013).

An outbreak of psittacine beak and feather disease (PBFD) in 2005 (Kundu *et al*. 2012) prompted the end of the intensive period of rescue, rear and release of individuals, a decision that was made to minimize any management‐mediated disease transmission. PBFD is known to substantially increase juvenile mortality in this and other species (Ritchie *et al*. 1989; Jones & Merton 2012). In particular, on Mauritius PBFD is suspected to have wiped out an entire reintroduction attempt in 2004/2005 when 32 out of 36 released individuals disappeared with clinical signs (MWF 1994‐2013; Tollington *et al*. 2013). Psittacine beak and feather disease is a condition characterized by feather dystrophy and immunosuppression, and although rare incidences had been recorded in this population prior to 2005 (Greenwood 1996), the disease was not considered a specific threat to the species' continued recovery. Although PBFD is potentially fatal, affected individuals commonly recover from acute clinical signs, but juveniles are known to be more susceptible (Todd 2000). The causative agent of PBFD, BFDV is one of the most common infections of parrots (Ritchie *et al*. 1989). It is a vertically and horizontally transmitted single‐stranded DNA circovirus, and many individuals are apparently able to tolerate infection without developing disease.

**Figure 1:**
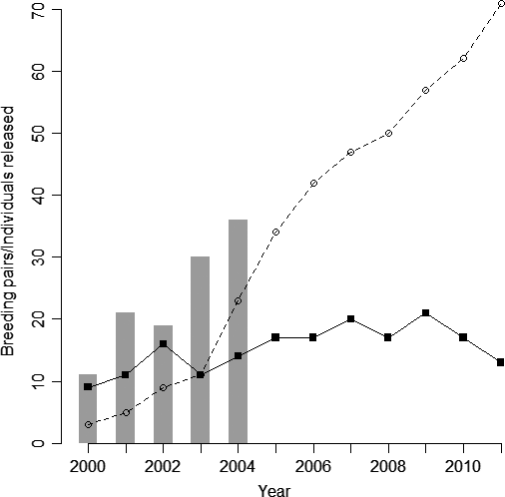
Total numbers of released individuals (grey bars) and breeding pairs per year during the 12‐year study period. Dashed lines and open circles represent breeding pairs which took supplementary food whilst solid lines and filled circles those which did not.

The 2005 disease outbreak was associated with a specific and novel isolate of BFDV, which emerged as a result of a mutation in the replication‐associated gene of the virus, and subsequently became fixed among the viruses infecting Mauritius parakeets (Kundu *et al*. 2012). The origin of this mutation is unknown but the timing of the outbreak coincided with the last release cohort of 36 hand‐reared individuals in 2004/2005. Only four individuals (11%) from this cohort are known to have survived to 2 years, and clinical signs were noted in many soon after release (MWF 1994‐2013). In contrast, of the 103 individuals which were released prior to this disease outbreak, at least 50 (49%) subsequently survived to enter the breeding population (MWF 1994‐2013). Counter‐intuitively, despite the outbreak of disease, marked by the sudden irruption of both captive and free‐living individuals of all ages presenting with clinical signs, the recovery of this once critically endangered species has continued unimpeded (Fig. 1). Furthermore, recent molecular work suggests perhaps surprisingly that this species has retained relatively high levels of neutral genetic diversity despite its historic bottleneck (Raisin *et al*. 2012; Tollington *et al*. 2013). For example, Tollington *et al*. (2013) found that the mean heterozygosity of the ninety known breeding individuals in 2010, across 16 microsatellite loci (0·64), was comparable to that of many widespread, non‐threatened and continental species, although a reduction in heterozygosity and allelic richness was observed over two decades across the programme. Total population size and the number of monitored breeding pairs continue to increase, and current (2013) estimates exceed 500 individuals and 95 known breeding pairs (MWF 1994‐2013).

That population growth in the Mauritius parakeet was seemingly unhindered by the confirmed outbreak of a novel and potentially lethal virus isolate raises questions concerning the true impacts of disease outbreak at the population level. Few studies are able to examine the sublethal effects of an immunosuppressive virus on breeding success, but doing so is entirely possible in the case of the Mauritius parakeet because of the existence of long‐term monitoring data. Collection of detailed breeding records began long before the disease outbreak occurred and were subsequently segmented into pre‐, during, and post‐disease outbreak phases and combined with data concerning individual conservation management histories and life histories of breeding birds. We explored the predictors of clutch size, hatch success and fledge success across a 12‐year study period and assessed the potentially negative impact of disease outbreak, the putatively positive impacts of supplementary feeding and the interactive effects on these three stages of productivity. We then investigated potential environmental, genetic and management‐related drivers of individual BFDV infection in nestlings post‐outbreak and tested the hypothesis that BFDV‐infected individuals would reveal reduced immune responses as a result of the immunosuppressive nature of the virus. We subsequently interpret our findings in the light of conventional wisdom regarding expected effects of disease outbreaks on small populations.

## Materials and methods

### Productivity

The study was conducted using productivity data collected over a period of 12 years, from 2000 to 2011 in the Black River Gorges National Park, Mauritius (20°23′S, 57°23′E). Mauritius parakeets form monogamous pair bonds which last for many years and typically produce a single clutch of eggs per year. A total of 601 monitored nesting attempts (where at least one egg was laid) by 131 different females were monitored between 2000 and 2011 (MWF 1994‐2013). Determinants of clutch size (number of eggs), hatch success and fledge success of hatched individuals (successes, failures), parameters of reproductive effort that are known to be affected by disease in birds (Norris & Evans 2000; Ardia 2005), were investigated separately with the following explanatory variables demonstrated to influence productivity and/or disease susceptibility: female age (as a quadratic term (Forslund & Pärt 1995)), standardized multilocus heterozygosity (MLH) (Whiteman *et al*. 2006; Tollington *et al*. 2013) and lay date of first egg (Hasselquist, Wasson & Winkler 2001) (number of days after September 1st). Additional predictor variables were disease phase (pre‐ (2000–2004), during (2005) and post‐disease (2006–2011) outbreak) and parental supplemental feeding (true or false). Secondly clutches, laid as a result of initial clutch or brood failure, were omitted from our analyses (*n* = 26).

### BFDV Infection

Infection status (coded as a binary response variable) was determined for nestlings between 2005 and 2009 (Kundu *et al*. 2012). We only included BFDV infection status of nestlings at 45 (± 3) days old (all individuals were known to successfully fledge at 50‐69 days old, mean 57, and are hereafter termed fledglings) to avoid any sampling bias associated with the relatively difficult capture of adults. Explanatory parameters selected as potential factors affecting viral susceptibility (Acevedo‐Whitehouse *et al*. 2003; Schmid‐Hempel 2003; Lee 2006; Robb *et al*. 2008) were as follows: nest type (natural cavity or artificial box), brood size at fledging, lay date, female age, supplemental feeding, hatch order, year as a categorical variable (2005–2009), gender and individual MLH (Tollington *et al*. 2013).

### Indices of Immune Function

The effects of environmental, genetic and morphological variables on immune function parameters were assessed for fledglings produced in 2009/2010 and 2010/2011. Individual innate humoral and cellular immune response was assessed using three methods: (i) the phytohaemagglutinin (PHA) challenge technique (Smits, Bortolotti & Tella 1999), (ii) the heterophil‐to‐lymphocyte (H:L) ratio (Campbell & Ellis 2006) and (iii) the haemolysis–haemagglutination assay (HL‐HA) (Matson, Ricklefs & Klasing 2005). Separate analyses were performed for each measured response variable. Explanatory variables included in these models were as previously stated with the inclusion of a further explanatory term (PHA challenged: true or false) to the models which investigated cellular (H:L ratio) and humoral (HL‐HA) indices of immune function. Hatch order was replaced by body mass and wing length to account for interbrood variation in growth rates.

### Statistical Procedure

We used generalized linear mixed models (GLMMs) and an information‐theoretic approach to model selection (Burnham & Anderson 2002; Whittingham *et al*. 2006) in order to identify predictors of breeding productivity, BFDV infection status and indices of immune function. Before model averaging, we restricted all model sets to ΔAIC_c_<4 in order to eliminate potentially implausible models with low AIC weights (Burnham & Anderson 2002; Bolker *et al*. 2009). Averaged parameter estimates (β), unconditional standards errors (SE), upper and lower confidence intervals (UCI, LCI) and relative variable importance factors (RI) are reported in summary results of GLMMs after model averaging. Where our restricted model set resulted in only one model at ΔAIC_c_<4, model averaging was not necessary and we applied likelihood ratio chi‐squared tests to test for significant parameters. Random effects were included in all models to account for spatial and temporal pseudoreplication, female identity and year in all productivity models, and nest identity for BFDV infection and immune response models. Productivity models included all first‐order explanatory variable interactions with ‘outbreak phase’. A Wilcoxon rank‐sum test was used to test for differences in numbers of fledglings produced per breeding attempt between those pairs which supplementary fed and those which did not. Where the response was complement‐mediated lysis (HL‐HA), zero‐inflated GLMMs (ZIGLMMs) were used (many individuals revealed a zero lysis reaction). All statistical procedures were performed using R (R Development Core Team 2012), and extended methods and statistical procedures can be found in the accompanying supplementary material.

## Results

### Productivity

**Figure 2:**
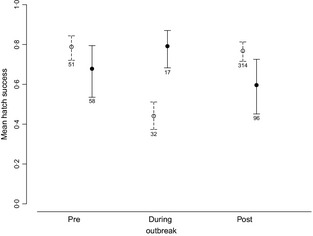
Mean hatch success (from fitted models) and 95% binomial confidence intervals of breeding attempts according to supplemental feeding pre‐, during and post‐disease outbreak revealing significantly reduced levels during the outbreak phase. Breeding pairs which took supplemental food are represented by dashed lines and open circles and sample sizes are shown.

Model averaging was not necessary to identify predictors of clutch size as only one model remained at ΔAIC_c_<4. Clutch size was significantly and positively associated with female age (β = 0·03, SE = 0·001, χ^2^ = 3·80, *P* < 0·001) and negatively with lay date (β = −0·01, SE = 0·002, χ^2^ = −6·75, *P* < 0·001). Hatch success was significantly associated with outbreak phase, and significantly and negatively associated with supplemental feeding, there was also a significant interaction between these two terms (Table 1). Hatch success during the outbreak phase was significantly reduced in comparison to pre‐ and post‐outbreak phases, but this decrease was only apparent among breeding attempts involving adults which took supplementary food (Table 1 and Fig. 2). No significant variation associated with hatch success was revealed among breeding attempts involving adults which did not take supplemental food. When we restricted our model to supplementary‐fed broods during the outbreak phase and included distance from nest site to supplementary feeding hoppers as an explanatory variable, we found no significant variation in hatch success (Table S1). Fledging success was significantly higher for nesting attempts involving supplementary‐fed parents, but no effect of outbreak was revealed (Table 1), and the number of fledglings per breeding attempt among supplemental pairs was significantly higher than that of non‐supplemental fed pairs (*P* < 0·001). Pairs that were supplementary fed produced on average 0·44 (± 0·20) more fledglings than pairs that did not supplementary feed. None of the other explanatory variables significantly predicted productivity. Figure S1 summarizes annual hatch and fledge success.

**Table 1:** Results of model averaged GLMMs fitted with binomial errors to investigate predictors of hatch and fledge success and interactions with supplemental feeding and disease outbreak between 2000 and 2011

Response	Predictor	β	SE	LCI	UCI	RI
Hatch success	(Intercept)	0·26	0·25	−0·23	0·74	
*N*_broods_ = 464	Supplemental feeding	−**2·24**	**0·59**	−**3·39**	−**1·09**	**1·00**
	Outbreak (pre)	**0·94**	**0·32**	**0·32**	**1·56**	**1·00**
	Outbreak (post)	**1·03**	**0·25**	**0·53**	**1·53**	**1·00**
	Female age	−0·08	0·31	−0·68	0·52	1·00
	Female MLH	−0·02	0·37	−0·75	0·70	1·00
	Lay date	−1·08	0·57	−2·19	0·03	1·00
	Supp:outbreak (pre)	**2·06**	**0·66**	**0·77**	**3·35**	**1·00**
	Supp:outbreak (post)	**2·80**	**0·59**	**1·64**	**3·96**	**1·00**
	Outbreak (pre):lay date	0·17	0·72	−1·25	1·59	0·43
	Outbreak (post):lay date	0·89	0·67	−0·43	2·20	0·43
	Outbreak (pre):female MLH	−0·44	0·71	−1·83	0·95	0·25
	Outbreak (post):female MLH	0·44	0·56	−0·66	1·54	0·25
	Outbreak (pre):female age	−0·09	0·77	−1·60	1·42	0·13
	Outbreak (post):female age	0·46	0·61	−0·73	1·64	0·13
Response	(Intercept)	1·61	0·34	0·95	2·27	
Fledge success	Supplemental feeding	**0·84**	**0·32**	**0·21**	**1·47**	**1·00**
*N*_broods_ = 464	Female age	0·19	0·24	−0·29	0·66	1·00
	Female MLH	−0·04	0·27	−0·57	0·48	1·00
	Lay date	−0·07	0·23	−0·52	0·39	1·00
	Outbreak (pre)	0·75	0·58	−0·39	1·88	0·28
	Outbreak (post)	0·16	0·53	−0·88	1·21	0·28

Significant explanatory parameters, where confidence intervals do not cross zero, are highlighted in bold. Estimates of random effects were 0·51 ± 0·71 and 0·14 ± 0·38 for ‘female ID’ and ‘year’, respectively, for hatch success and 0·81 ± 0·90 and 0·15 ± 0·39 for fledge success.

### Prevalence and Predictors of BFDV Infection

Between 2005 and 2009, 425 fledglings from 223 broods were assessed for BFDV infection (Fig. S2), none of which presented clinical signs which are usually only apparent in this species after the post‐fledging moult. A total of 83 individuals (19·5%) from 68 broods showed a positive result (mean prevalence within positive broods 61%). Neither female ID nor nest ID explained significant variation in infection status (intraclass correlations (ICC), Female: ICC = −0·01, 95% CI = −0·08 to 0·08, Nest ID: ICC = 0·15, 95% CI = −0·04 to 0·31). The only significant predictor of individual infection status was year; prevalence was significantly lower in 2006, 2008 and 2009 than in the outbreak year 2005 (Table S2).

### Immune Function Variables

Status of BFDV infection was determined for 132 fledglings from 2009 with 31 individuals revealing infection, 60 of these individuals were also subjected to the PHA challenge. The magnitude of the PHA response was not influenced by BFDV infection (BFDV negative: *n* = 46, mean PHA response = 0·55 mm ± 0·41; BFDV positive: *n* = 14, mean PHA response = 0·57 mm ± 0·35. *t* = 0·16, d.f = 24·79, *P* = 0·87). Those individuals which were experimentally challenged with PHA revealed a significantly higher H:L ratio than those that were not, but infection with BFDV provoked no significant response in any of the immune function indices (Table S3). There were no significant effects of an interaction between individual BFDV status and PHA challenge on any of the immune function measures (all *P* > 0·17).

Very few environmental, genetic and management‐related factors were significantly associated with our indices of immune function. The only term which explained significant variation in magnitude of response to PHA was year (Table S4). Individuals exposed to PHA revealed an increased H:L ratio compared to those not exposed to the experimental immunostimulation, but no other parameter was associated with H:L ratio (Table S4). Exposure to PHA also produced an increased lysis reaction within the HL‐HA test and male individuals reacted more strongly than females (Table S4).

## Discussion

Establishing the resilience of natural populations to disease outbreaks and their associated sublethal effects is challenging without detailed life‐history information spanning an outbreak such as that documented for the Mauritius parakeet. Furthermore, the emergence of infectious disease among small populations can be regarded as disastrous by conservation biologists, who are then faced with evaluating the impact that interventive strategies might have on disease effects. Our study has demonstrated that an epidemic outbreak of an endemic disease does not necessarily lead to deleterious effects on population growth (number of breeding pairs) and may only have short‐lived, negative consequences. More importantly, we have illustrated how the effects of disease outbreak in a recovering, bottlenecked and once critically endangered population, ordinarily associated with increased susceptibility to disease (Lyles & Dobson 1993; Smith, Acevedo‐Whitehouse & Pedersen 2009), appear to have been surprisingly benign.

Supplemental feeding is often used to aid the recovery of threatened species or to support populations of garden birds and is frequently justified by the assumption that it can increase individual fitness and thus population growth where availability of natural food is believed to be a limiting factor (Newton 1998; Jones & Merton 2012). Food provisioning is, however, recognized as an agent of ecological change, the direct and indirect effects of which can have both positive and negative effects on the target species and wider ecological interactions (Robb *et al*. 2008; Oro *et al*. 2013). We have demonstrated that parakeets which took supplemental food generally fledged a higher proportion of chicks than pairs which did not use this resource. Furthermore, our results suggest that hatching success among non‐fed breeding pairs is more variable than that among fed pairs (Fig. S1), which may be attributed to the contrast between annual fluctuations in natural food availability and the consistency of supplemental provisioning. Supplemental feeding therefore appears to be beneficial by (i) enabling breeding pairs to fledge more offspring and (ii) by mitigating the negative effects of fluctuating natural food resources. But our results also hint at the less obvious and indirect consequences on ecological interactions with disease.

Food provisioning may exacerbate the subtle, negative effects of infectious disease on elements of reproductive fitness during an outbreak. An increase in egg infertility or embryo mortality (we could not determine the exact effect) coincided with the outbreak of a novel BFDV isolate, but the effects were only apparent among the proportion of the population of breeding pairs which used supplemental food. This result suggests that an association with the supplemental feeding hoppers in some way facilitated an increase in viral transmission between individuals, but our infection results do not support this theory; infection prevalence was not significantly higher among supplementary‐fed birds than non‐supplementary fed.

### Why was Reduced Hatch Success During the Outbreak Only Apparent Among Supplementary‐Fed Breeding Pairs?

We cannot exclude the unlikely possibility that a change in quality of the commercially available parrot food during this year was a contributing factor. A reduction in fertility and hatch success could of course be associated with, for example, genetic effects (which would be apparent in multiple years) or environmental events such as cold periods which would have affected the whole population. We can think of no other factor (besides the viral outbreak) which would only manifest in one sector of a breeding population of a bird species which form monogamous pair bonds lasting for many years and which display high breeding‐site fidelity.

Why then, were the effects of this outbreak only apparent in fed birds? Perhaps there is a spatial element? For example, Tollington *et al*. (2013) suggest that this species exists in three putative subpopulations, of which two (those furthest apart) are provided with supplemental food, but individuals from all three populations take supplementary food. However, the short‐lived nature of the impacts of the outbreak that we detected and the fact that these subpopulations also include non‐fed breeding pairs do not support a spatial explanation. The infection results in this study were based on a traditional PCR assay of a single blood sample per individual which returns a binary (true or false) infection status determined by visualizing the presence or absence of a PCR product on agarose gel. Our results may therefore be explained by considering how these infection results ought to be interpreted; especially, considering that prevalence in 2007 was no different from that in the outbreak year. Infection prevalence is a poor indicator of the impact of disease on a population as individuals can be *infected* without being *affected* (Lyles & Dobson 1993; Cunningham 1996), and the diagnostic method used here does not distinguish clinical from subclinical infections. Furthermore, traditional methods of detecting infection status do not consider infection intensity or load (Scott 1988). BFDV can exist as a latent or productive infection, and therefore, it does not necessarily follow that an individual which tests positive for BFDV will develop PBFD (Rahaus & Wolff 2003). Consequently, our results may be explained by infection type and/or intensity; it is likely that those individuals which use supplemental food are, for some reason, more *affected* by the virus. This explanation may be true because supplemental feeding alters an aspect of their natural behaviour, thereby acting as an agent of ecological change. For example, attracting large numbers of birds to feeding hoppers can lead to locally high population densities which increases contact rates among individuals and facilitates increased viral transmission (Bradley & Altizer 2007). Supplementary‐fed individuals may have been more affected by the outbreak of a novel viral isolate simply because they came into contact with it more often than non‐supplementary‐fed birds as a result of increased density, triggering a physiological trade‐off between immune system response and reproductive effort (Norris & Evans 2000) as individual viral load reached a threshold level. Somewhat surprisingly, these negative effects were short‐lived and hatch success rapidly returned to pre‐outbreak levels which may reflect the nature of the selective sweep of this viral isolate and the rapid replacement by a less virulent genotype (Kundu *et al*. 2012). Nevertheless, our study has clearly shown that supplemental feeding can have differential and often beneficial effects at varying stages of brood productivity, but disease outbreak negated some of these benefits by reducing hatch success to levels below that of breeding pairs which did not take supplemental food. One alternative interpretation is that reduced hatching success during the outbreak led to higher fledging success due to reduced sibling competition, but this expectation is not evident from year‐on‐year trends (see Fig. S1).

### Interpreting Infection Status in Studies of Disease Ecology

We did not identify any genetic, environmental or management‐related factors which predicted individual BFDV infection status. Furthermore, infection status was not recognizably associated with any depression of immune response either in those individuals also experimentally challenged with PHA or those which were not. This result is surprising given that BFDV is an immunosuppressive virus (Todd 2000). Species which have endured a population bottleneck event might be expected to reveal immunological impairment as a result of genetic impoverishment associated with small, fragmented populations (Whiteman *et al*. 2006). This immunological impairment potentially manifests as a lack of response to an immune challenge (Spielman *et al*. 2004) which may be the case for the Mauritius parakeet, although we recognize that the methods of assessing variables of immune response used in this study are regarded by some as limited in their sensitivity (Demas *et al*. 2011). On the contrary, we demonstrated how experimental immunostimulation with PHA provoked an innate response among fledgling parakeets, confirming the existence of a functioning reactive immune system. Studies of neutral genetic diversity in this species (Tollington *et al*. 2013) suggest that it has in fact lost little diversity as a result of a population bottleneck but characterizing quantitative loci under strong selection such as those involved in immunity (e.g. major histocompatibility complex or toll‐like receptors) may provide important missing pieces of the puzzle. In a similar study, Ortiz‐Catedral (2010) found that BFDV infection resulted in reduced PHA‐induced swelling, but we found no such variation in magnitude of swelling between BFDV‐infected and non‐infected individuals. These results perhaps call into question the significance of interpreting our measure of individual infection status from a single point in time which highlights the difficulty in identifying the effects of disease in free‐living populations and the importance of systematic post‐release monitoring of reintroduced populations (Armstrong & Seddon 2008; Sutherland *et al*. 2010). Identifying the life‐history stages that affect productivity in reintroduced populations can only be achieved with long‐term monitoring data which, as demonstrated by this study, often produce unforeseen and unpredictable results.

Our results highlight the importance of long‐term systematic post‐release monitoring in conservation reintroduction attempts and their value in identifying the subtle, sublethal effects of disease outbreak. In the reintroduced Mauritius parakeet, hatching success of breeding pairs which used supplemental food was significantly reduced during a disease outbreak and furthermore was reduced below that of breeding pairs which did not take supplemental food. Pre‐outbreak levels of hatch success returned surprisingly quickly after the outbreak had subsided. Although intensive management can exacerbate the negative effects of disease outbreak, the impact can be short‐lived, a positive outlook for conservation managers.

## Data accessibility

Data are available from the Dryad Digital Repository: http://dx.doi.org/10.5061/dryad.83r6m (Tollington *et al*. 2015).

## Supporting information


**Data S1**. Methods.
**Fig. S1**. Twelve year productivity data for Mauritius parakeets.
**Fig. S2**. Prevalence of BFDV over the intensive sampling period.
**Table S1**. Results of model averaged binomial GLM to predict hatch success among supplementary fed broods during the outbreak phase.
**Table S2.** Predictors of individual BFDV infection status.
**Table S3.** Effects of PHA challenge and BFDV infection on variables of immune function for fledglings from 2009/10 season.
**Table S4.** Results of model‐averaged GLMMs (and ZIGLMM where response was lysis) to identify predictors of immune function indices for fledglings produced during the two breeding seasons 2009/10 and 2010/11.
