# Supplementary material for: Detailed monitoring of a small but recovering population reveals sublethal effects of disease and unexpected interactions with supplemental feeding
Source: J Anim Ecol. 2015 Mar 9;84(4):969–77. doi: 10.1111/1365-2656.12348 (PMC5098166; doi:10.1111/1365-2656.12348)
Supplement: Supplementary file 1 — Data S1. Methods. Fig. S1. Twelve year productivity data for Mauritius parakeets. Fig. S2. Prevalence of BFDV over the intensive sampling period. Table S1. Results of model averaged binomial GLM to predict hatch success among supplementary fed broods during the outbreak phase. Table S2. Predictors of individual BFDV infection status. Table S3. Effects of PHA challenge and BFDV infection on variables of immune function for fledglings from 2009/10 season. Table S4. Results of model‐averaged GLMMs (and ZIGLMM where response was lysis) to identify predictors of immune function indices for fledglings produced during the two breeding seasons 2009/10 and 2010/11. [file JANE-84-969-s001.docx]

**Detailed monitoring of a conservation reintroduction reveals sublethal effects of disease and unexpected interactions with supplemental feeding**

Electronic supplementary material

Supplementary methods

**PHA protocol and blood sample processing**

The PHA challenge technique is known to provoke a T-cell mediated immune reaction in birds involving both innate and adaptive components of the immune system [1-2]. Despite uncertainty regarding its precise effects and interpretation of the induced response, it is widely used in studies of avian immune function to experimentally provoke an immune reaction [e.g. 3, 4]. Twenty microlitres of 5:1 PHA (Sigma-Aldrich L8754) in phosphate buffer (PBS; Oxoid, Dulbecco A–BR0014G) solution was administered intradermally to the right wing-web of each nestling. At least three measurements of patagium thickness at the injection site were taken to the nearest 0.01mm using a pressure-sensitive micrometer (Silverline 282378) prior to PHA exposure and the average of these measurements was taken as the pre-injection thickness. After a period of 24 hours (±2 hrs) the tissue thickness at the injection site was measured in a similar manner and the response to the PHA challenge was recorded as the average post-injection measurement minus the average pre-injection measurement. Blood was collected from PHA-challenged individuals during the post-injection measurement procedure. The reasons for collecting blood samples from these individuals after the PHA challenge were twofold; this reduced the handling time of each nestling during the same nest site visit and also gave us the opportunity of studying the physiological response to PHA by comparing cellular and humoral variables of immune function between those individuals which had received the PHA challenge and those which had not.

Each blood sample was divided three ways to facilitate a variety of subsequent analyses:

1. Fresh, whole blood was taken directly from the syringe and at least two blood slides were made using the wedge method [5-6]. These were air dried and fixed in 100% methanol for staining and leukocyte profiling.

2. Two to three drops of whole blood were stored in absolute ethanol to enable DNA extraction for genotyping and disease profiling.

3. The remainder of the blood, ~500µl was transferred to a heparinised collection tube (Teklab H1230) and gently agitated to prevent coagulation to facilitate the assessment of humoral immune function by natural antibodies (NAbs) and complement mediated lysis using the haemolysis-haemagglutination (HL-HA) technique [7].

Samples were transferred back to the field laboratory where the blood smears were stained with Leishman's stain solution (Fisher PB05) following the manufacturer's guidelines. The remainder of each blood sample was centrifuged at 8000rpm for five minutes before plasma was extracted and stored at -80ºC until it could be transported back to the UK.

**Heterophil to lymphocyte ratio**

Examination of the leukocyte profile from blood slides is also widely used in field studies that investigate avian immune defence [reviewed in 8]. Previously used to infer levels of stress [9-10] concentrations of different leukocytes reflect the stimulation of both innate and acquired immune defences [11] and the assessment of the heterophil to lymphocyte ratio (H:L) in particular has been associated with, for example, body condition [12], parasite infection [13], experimental immunological stimulation [14], and genetic diversity [15].

**General haemolysis-haemagglutination assay**

Assessment of the B-lymphocyte governed humoral immunity is facilitated by the examination of serum proteins including antibodies and complement. Natural antibody levels and complement mediated lysis of foreign antigens were assessed using the general haemolysis-haemagglutination assay (HL-HA) [7] for each nestling produced between 2009 and 2010. The assay was performed following the methods of Matson et al., [7] with some modifications. Twenty-five microlitres of 1.0 M PBS (Oxoid, Dulbecco A – BR0014G) were added to all wells in columns 2-12 of a 96-well, U-bottom microplate (Sterilin 612U96). Twelve and a half microlitres of eight individual plasma samples were then added to columns 1 and 2 and a multi-channel pipette was used to serially dilute the plasma solution between columns 2 and 11 resulting in dilutions ranging from 1:3 in column 2 to 1:59049 in column 11. Column 1 was used as a positive control given that it contained only plasma and column 12 as a negative control as it contained only PBS. In every third plate, a control of chicken plasma was used alongside that of parakeet nestlings to ensure assay consistency. Twelve and a half microlitres of a freshly prepared solution of 1% rabbit red blood cells (RRBCs) (Harlan Laboratories UK) in PBS were then added to each well of the microplate to initiate the assay. Plates were then incubated at 37ºC for 90 minutes before being scanned with a top-lit flatbed scanner in order to visualise and score the agglutination of RRBCs by natural antibodies. After a further 60 minutes at room temperature the plates were scanned for a second time in order to score the complement mediated lysis of RRBCs. Each individual nestling was therefore given two scores, one for agglutination and one for lysis, both of which corresponded to the last cell of the microplate in which each reaction was observed.

**Statistical procedure**

All statistical analyses were performed within the programming environment R [16]. Extensive exploratory analyses including graphical inspection, correlation matrices and simple bivariate tests were performed and revealed a low number of influencing, outlying data points. The results presented here do not include any models where these data were found to significantly affect final model outcomes; they were eliminated and the models repeated. Variance inflation factors (VIFs) were used to assess collinearity of all explanatory parameters [17] and no parameters with a VIF >2.4 were included in any of our models. Generalised linear mixed models (GLMMs) and zero-inflated GLMMs (ZIGLMMs) were applied using appropriate error structures in the R packages lme4 [18] and glmmADMB [19] respectively. All input variables were scaled to a mean of zero and a standard deviation of 0.5 following Gelman [20], to aid comparative interpretation of model averaged coefficients.

We adopted an information theoretic approach to model selection using the R package MuMIn [21] to evaluate all candidate models in a given set based on AIC_c_ (AIC adjusted for small sample size) and Akaike weights. Final model sets were restricted before model averaging to all models where ΔAIC_c_ < 4 in order to eliminate potentially implausible models with low AIC weights [21-24]. Model averaging was then applied to the reduced model set to compute a weighted average of parameter estimates and their associated standard errors (SE). The relative importance of explanatory variables were then calculated by summing the Akaike weights across all models in which the variable was present resulting in an estimate of probability that the variable of interest features in the best model.

**Supplementary figures and tables**


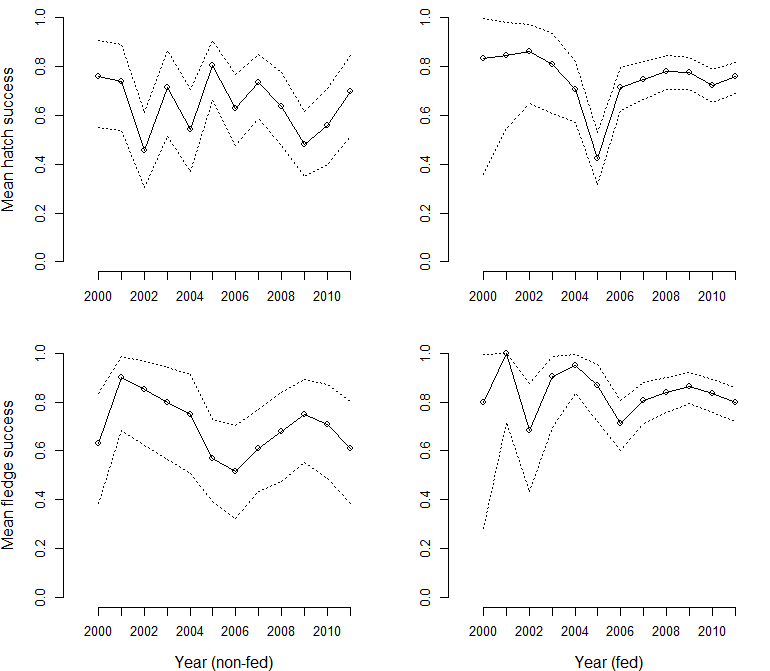
Figure S1 Twelve year productivity data for Mauritius parakeets. Means and 95% binomial confidence intervals of hatch and fledge success are plotted using raw data for each year with supplementary fed pairs on the right and non-supplementary fed pairs on the left.


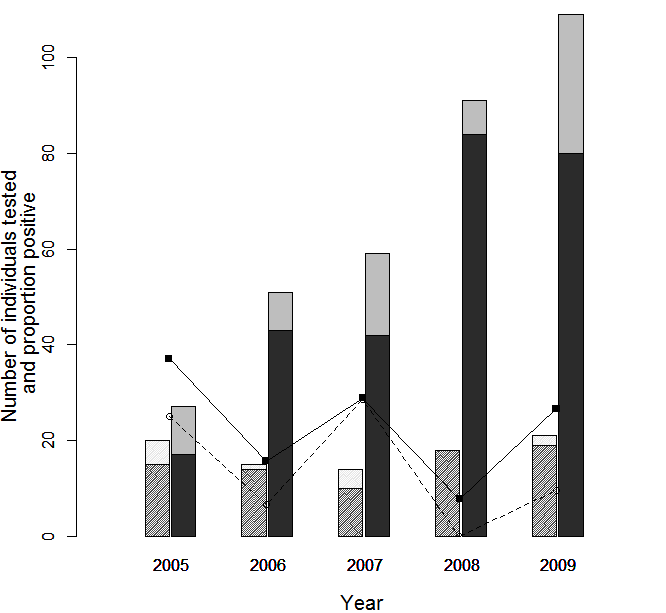


Figure S2 Prevalence of BFDV over the intensive sampling period. Column heights represent the total number of individuals tested in each year arranged by supplementary fed broods (solid bars) and non-supplementary fed broods (hatched bars). Column stacks represent the number of individuals which returned a negative result (dark) and those which tested positive (light). Points and lines represent the proportion of all individuals tested which returned a positive result, separated by supplemental fed (closed, solid) and non-supplemental fed (open, dashed).

Table S1 Results of model averaged binomial GLM to predict hatch success among supplementary fed broods during the outbreak phase. Averaged parameter estimates (β) unconditional standards errors (SE), upper and lower confidence intervals (UCI, LCI) and relative variable importance factors (RI) are reported.

| Response | Predictor | β | SE | LCI | UCI | RI |
| --- | --- | --- | --- | --- | --- | --- |
| Hatch success | (Intercept) | -0.62 | 0.50 | -1.59 | 0.36 |  |
| N_broods_ = 30 | Female MLH | -0.68 | 0.60 | -1.85 | 0.50 | 0.78 |
|  | Hopper distance | -1.52 | 0.88 | -3.25 | 0.21 | 0.41 |
|  | Lay date | -0.82 | 0.73 | -2.25 | 0.61 | 0.31 |
|  | Female age | -0.66 | 0.97 | -2.57 | 1.24 | 0.27 |

Table S2 Predictors of individual BFDV infection status. Results of model averaged GLMMs using a binomial distribution and logit link with infection status as a binary response variable, nest ID was included as a random factor. Significant predictors (where confidence intervals do not cross zero) are bold. Relative importance factors (RI) represent an estimate of probability that the variable of interest features in the best model.

|  | Predictor | β | SE | LCI | UCI | RI |
| --- | --- | --- | --- | --- | --- | --- |
| BFDV infection | (Intercept) | -0.31 | 0.38 | -1.05 | 0.43 |  |
| N_individuals_ = 380 | **Year 2006** | **-1.30** | **0.56** | **-2.40** | **-0.20** | **1.00** |
| N_broods_ = 212 | Year 2007 | -0.74 | 0.49 | -1.70 | 0.22 | 1.00 |
|  | **Year 2008** | **-2.52** | **0.57** | **-3.64** | **-1.40** | **1.00** |
|  | **Year 2009** | **-1.04** | **0.45** | **-1.92** | **-0.16** | **1.00** |
|  | Nest type (c) | -0.64 | 0.37 | -1.37 | 0.08 | 0.68 |
|  | Brood size | 0.54 | 0.33 | -0.10 | 1.19 | 0.64 |
|  | MLH | -0.46 | 0.29 | -1.03 | 0.10 | 0.60 |
|  | Supp (true) | 0.57 | 0.43 | -0.28 | 1.41 | 0.44 |
|  | Female age | 0.30 | 0.32 | -0.33 | 0.94 | 0.28 |
|  | Hatch order | -0.12 | 0.31 | -0.74 | 0.49 | 0.17 |
|  | Sex | -0.10 | 0.28 | -0.65 | 0.46 | 0.14 |

Table S3 Effects of PHA challenge and BFDV infection on variables of immune function for fledglings from 2009/10 season. Where responses were H:L and agglutination GLMMs were applied using a Gaussian error structure. NestID was included in all models as a random effect, H:L was log-transformed to achieve residual normality and significance of model terms was derived using likelihood ratio chi-squared tests. Effects on lysis were investigated using ZIGLMMs and poisson errors.

| Response | Predictor | β | SE | χ^2^ (z) | P |
| --- | --- | --- | --- | --- | --- |
| H:L | **Challenged** | **0.56** | **0.17** | **11.02** | **0.00** |
| N_individuals_ = 146 | BFDV | 0.09 | 0.19 | 0.24 | 0.62 |
| N_broods_ = 56 | Interaction | -0.39 | 0.29 | 1.87 | 0.17 |
| Agglutination | Challenged | -0.32 | 0.24 | 1.76 | 0.18 |
| N_individuals_ = 120 | BFDV | -0.54 | 0.27 | 4.01 | 0.05 |
| N_broods_ = 58 | Interaction | 0.48 | 0.41 | 1.19 | 0.27 |
| Lysis | Challenged | 0.61 | 0.36 | (1.69) | 0.09 |
| N_individuals_ = 120 | BFDV | 0.60 | 0.48 | (1.26) | 0.21 |
| N_broods_ = 58 | Interaction | -1.00 | 0.74 | (-1.35) | 0.18 |

Table S4. Results of model-averaged GLMMs (and ZIGLMM where response was lysis) to identify predictors of immune function indices for fledglings produced during the two breeding seasons 2009/10 and 2010/11. Only those explanatory predictors which remained in the final averaged model set are included in this table. Gaussian error structures were applied to all models with the exception of that investigating lysis (poisson errors). NestID was included as a random factor in all models. Significant predictors, where confidence intervals do not cross zero, are shown in bold. Relative importance factors (RI) represent an estimate of probability that the variable of interest features in the best model.

| Immune component | Explanatory variable | β | SE | LCI | UCI | RI |
| --- | --- | --- | --- | --- | --- | --- |
| PHA | Intercept | 0.60 | 0.12 | 0.36 | 0.85 | N/A |
| N = 116, 53 | **Year 2010** | **0.33** | **0.08** | **0.18** | **0.47** | **1.00** |
|  | SH | -0.22 | 0.16 | -0.53 | 0.10 | 0.21 |
|  | Supp (true) | -0.13 | 0.10 | -0.32 | 0.07 | 0.12 |
| H:L | Intercept | -0.51 | 0.05 | -0.61 | -0.40 | N/A |
| N = 212, 102 | **Challenged (true)** | **0.43** | **0.10** | **0.23** | **0.63** | **1.00** |
|  | Fledglings | 0.13 | 0.10 | -0.06 | 0.33 | 0.13 |
|  | Year 2010 | 0.13 | 0.10 | -0.07 | 0.33 | 0.13 |
|  | Supp (true) | 0.12 | 0.11 | -0.10 | 0.34 | 0.11 |
| Lysis | Intercept | -2.06 | 0.46 | -2.96 | -1.16 | N/A |
| N = 174, 94 | **Challenged (true)** | **1.54** | **0.47** | **0.61** | **2.47** | **1.00** |
|  | **Sex (male)** | **1.27** | **0.48** | **0.32** | **2.21** | **1.00** |
|  | **Challenged*Sex** | **-1.15** | **0.53** | **-2.19** | **-0.12** | **0.97** |
|  | **Year (2010)** | **0.45** | **0.22** | **0.02** | **0.89** | **0.84** |
|  | Supp (true) | -0.24 | 0.26 | -0.74 | 0.26 | 0.36 |
|  | Body mass | -0.26 | 0.22 | -0.70 | 0.18 | 0.35 |
|  | Wing length | -0.17 | 0.21 | -0.59 | 0.25 | 0.25 |
|  | Dam age | -0.05 | 0.21 | -0.46 | 0.37 | 0.13 |
|  | MLH | 0.01 | 0.21 | -0.39 | 0.42 | 0.13 |
| Agglutination | Intercept | 7.77 | 0.10 | 7.59 | 7.96 | N/A |
| N = 174, 94 | **Year (2010)** | **0.42** | **0.19** | **0.05** | **0.80** | **0.74** |
|  | Supp (true) | -0.30 | 0.21 | -0.71 | 0.10 | 0.24 |
|  | Body mass | 0.20 | 0.17 | -0.13 | 0.53 | 0.08 |
|  | Challenged | 0.07 | 0.19 | -0.30 | 0.44 | 0.05 |
|  | Dam age | -0.06 | 0.19 | -0.43 | 0.31 | 0.05 |
|  | Sex (male) | 0.10 | 0.15 | -0.20 | 0.39 | 0.05 |
|  | Wing length | 0.04 | 0.16 | -0.27 | 0.35 | 0.04 |
|  | MLH | 0.00 | 0.16 | -0.32 | 0.32 | 0.04 |

**Extended bibliography**

1. Martin L.B., Han P., Lewittes J., Kuhlman J.R., Klasing K.C., Wikelski M. 2006 Phytohemagglutinin-induced skin swelling in birds: histological support for a classic immunoecological technique. *Functional Ecology* **20**(2), 290-299.

2. Tella J.L., Lemus J.A., Carrete M., Blanco G. 2008 The PHA Test Reflects Acquired T-Cell Mediated Immunocompetence in Birds. *PLoS ONE* **3(9)**(9), e3295.

3. Heber S., Varsani A., Kuhn S., Girg A., Kempenaers B., Briskie J. 2013 The genetic rescue of two bottlenecked South Island robin populations using translocations of inbred donors. *Proceedings of the Royal Society B: Biological Sciences* **280**(1752).

4. Palacios M.G., Winkler D.W., Klasing K.C., Hasselquist D., Vleck C.M. 2011 Consequences of immune system aging in nature: a study of immunosenescence costs in free-living Tree Swallows. *Ecology* **92**(4), 952-966.

5. Campbell T.W., Ellis C.K. 2006 *Avian and Exotic Animal Hematology and Cytology, 3rd Edition*, Blackwell Science Ltd.

6. Clark P., Boardman W., Raidal S. 2009 *Atlas of clinical avian hematology*, Blackwell Publications.

7. Matson K.D., Ricklefs R.E., Klasing K.C. 2005 A hemolysis–hemagglutination assay for characterizing constitutive innate humoral immunity in wild and domestic birds. *Developmental and Comparative Immunology* **29**(3), 275-286.

8. Davis A., Maney D., Maerz J. 2008 The use of leukocyte profiles to measure stress in vertebrates: a review for ecologists. *Functional Ecology* **22**, 760-772.

9. Groombridge J.J., Massey J.G., Bruch J.C., Malcolm T.R., Brosius C.N., Okada M.M., Sparklin B. 2004 Evaluating stress in a Hawaiian honeycreeper, Paroreomyza montana, following translocation. *Journal of Field Ornithology* **75**(2), 183-187.

10. Garamszegi L.Z., Merino S., Török J., Eens M., Martínez J. 2006 Indicators of physiological stress and the elaboration of sexual traits in the collared flycatcher. *Behavioral Ecology* **17**(3), 399-404.

11. Feldman B.F., Zinkl J.G., Schalm O.W. 2000 *Schalm's veterinary hematology*, Wiley-Blackwell.

12. Palacios M.G., Cunnick J.E., Vleck D., Vleck C.M. 2009 Ontogeny of innate and adaptive immune defense components in free-living tree swallows, Tachycineta bicolor. *Developmental and Comparative Immunology* **33**(4), 456-463.

13. Gangoso L., Grande J.M., Lemus J.A., Blanco G., Grande J., Donázar J.A. 2009 Susceptibility to infection and immune response in insular and continental populations of egyptian vulture: implications for conservation. *PloS ONE* **4(7)**(7), e6333.

14. Sarv T., Hõrak P. 2009 Phytohaemagglutinin injection has a long-lasting effect on immune cells. *Journal of Avian Biology* **40**(5), 569-571.

15. Hale K.A., Briskie J.V. 2007 Decreased immunocompetence in a severely bottlenecked population of an endemic New Zealand bird. *Animal Conservation* **10**(1), 2-10.

16. R Development Core Team. 2012 R: A language and environment for statistical computing. (Vienna, Austria, R Foundation for statistical computing.

17. Zuur A.F., Ieno E.N., Walker N.J., Saveliev A.A., Smith G.M. 2009 *Mixed effects models and extensions in ecology with R*, Springer Verlag.

18. Bates D., Maechler M., Dai B. 2010 lme4: linear mixed-effects models using S4 classes. R package version 0.999375-33. R Foundation for Statistical Computing. Vienna, Austria. *CRAN R-project org/packageplme4*.

19. Skaug H., Fournier D., Nielsen A. 2008 glmmADMB: Generalized Linear Mixed Models Using AD Model Builder. (R package version 0.3. Available at <http://glmmadmb>. r-forge. r-project. org.

20. Gelman A. 2008 Scaling regression inputs by dividing by two standard deviations. *Statistics in medicine* **27**(15), 2865-2873.

21. Bartoń K. 2012 MuMIn: multi-model inference. *R package version* **1.2**, .

22. Burnham K.P., Anderson D.R. 2002 *Model selection and multimodel inference: a practical information-theoretic approach*, Springer Verlag.

23. Bolker B.M., Brooks M.E., Clark C.J., Geange S.W., Poulsen J.R., Stevens M.H.H., White J.S.S. 2009 Generalized linear mixed models: a practical guide for ecology and evolution. *Trends in Ecology & Evolution* **24**(3), 127-135.

24. Grueber C.E., Nakagawa S., Laws R.J., Jamieson I.G. 2011 Multimodel inference in ecology and evolution: challenges and solutions. *Journal of Evolutionary Biology* **24**(4), 699-711.
